# Beyond barriers: a qualitative evidence synthesis protocol of South Asian involvement in mental health research in high-income countries

**DOI:** 10.1136/bmjopen-2025-109567

**Published:** 2026-02-12

**Authors:** Nilam Akhtar Khan, Peter A Coventry, Habiba Ali, Helen Fulbright, Jennifer V E Brown

**Affiliations:** 1 Department of Health Sciences, University of York, York, UK; 2 School of Nursing and Public Health, Manchester Metropolitan University, Manchester, UK; 3 Centre for Reviews and Dissemination, University of York, York, UK; 4 School of Medicine and Population Health, The University of Sheffield, Sheffield, UK

**Keywords:** MENTAL HEALTH, QUALITATIVE RESEARCH, Health Equity, Health policy, Protocols & guidelines

## Abstract

**Abstract:**

**Introduction:**

There is a recognised need to provide equity in healthcare through inclusion of ethnic minorities in research. South Asians, the largest ethnic minority group in the UK, experience high levels of mental illness, often against the backdrop of socioeconomic deprivation and discrimination. The research community recognises that South Asian communities are often excluded from health research. Efforts have been made to understand the barriers and facilitators to their participation. However, the participation of individuals at the intersection of mental ill health and South Asian ethnicity remains understudied. This is a protocol for the synthesis of qualitative evidence from mental health research studies about participation, motivation and barriers to recruitment of South Asians in high-income countries.

**Methods and analysis:**

We will search 10 databases for qualitative evidence on participation of South Asian individuals in mental health research studies in high-income countries: MEDLINE, EMBASE, PsycINFO, Health Management Information Consortium, Social Policy and Practice, Cochrane Database of Systematic Reviews, Cochrane Central Register of Controlled Trials, Social Sciences Citation Index, Science Citation Index Expanded and ProQuest Dissertations and Theses Citation Index. Search terms for the following five concepts will be used: (1) South Asia, (2) ethnic minority, (3) mental health conditions, (4) barriers and facilitators to participation and (5) high-income countries. No date restriction will be applied to the search. Searches will be limited to studies in the English language. Study selection and data extraction will be performed by two researchers independently, using Covidence. Demographic data, thematic outputs and salient discussion points will be extracted from final full-text inclusions and entered into NVivo for coding. Meta-ethnographic approaches using first and second-order constructs from included studies will be used to form third-order constructs. This synthesis will generate new knowledge regarding the intersectionality of mental health and South Asian ethnicity.

**Ethics and dissemination:**

Ethical approval is not required as this study is a synthesis of published data. This review will provide new knowledge regarding the requirements for researchers and practitioners to advance the involvement of South Asian populations in mental health research. This will undoubtedly enhance equity in the long term, reduce the burden of mental disorders and enable the provision of more effective mental health care for South Asian communities.

**PROSPERO registration number:**

CRD42025626382.

**STRENGTHS AND LIMITATIONS OF THIS STUDY**

- The qualitative review will use meta-ethnography approaches to generate new knowledge on participation of South Asian communities in mental health research.
- Two of the review authors who are of South Asian backgrounds will be involved in the whole process of data extraction to interpretation, based on a sociocultural lens.
- The review will extract data from mixed-methods studies, and omission of quantitative outcomes may skew the overall understanding of the subject matter.
- It is thought that the qualitative findings are few in this area and will therefore provide novel insights.

## Introduction

 Participation in research not only enhances scientific knowledge but also brings about significant benefits for society. These benefits include improved understanding of health needs which in turn can pave the way for health innovation, equity and generalisable knowledge that benefits all, including groups that have historically been excluded from research. Individual motivations to partake in research may include altruism,^1^,^3^ the need for access to services or opportunities to express concerns about care. Marginalised groups, such as ethnic minority populations,^4^ tend to be motivated by knowledge enhancement and empowerment, as tangible consequences of research participation.^5^ At a societal level, research may enhance access to care and reduce the risks faced by minority groups in particular.^5 6^

Current debates in decolonial and equity research highlight that under-representation is not merely a methodological oversight but a form of epistemic exclusion.^7 8^ By consistently failing to include diverse voices, the research community risks perpetuating a narrow understanding of mental health that privileges dominant cultural norms.^9^ Addressing this is not only a matter of recruitment targets but an ethical and epistemic imperative to ensure that knowledge production accurately reflects the lived realities of South Asian communities.

South Asians, defined by UNICEF as individuals from Bangladesh, India, Maldives, Nepal, Pakistan and Sri Lanka, constitute the largest ethnic minority group in the UK (9.3%, 5.5 million, Department of Health census, 2021).^10^ In the UK, South Asians are overwhelmingly represented by people originating from India, Pakistan and Bangladesh, so UK figures mainly refer to these three groups.^11^ The UK South Asian population also experiences higher levels of mental illness compared with their white British counterparts.^12^,^14^ Discrimination and socioeconomic disadvantages experienced by members of South Asian communities may further compound psychological distress. Added to this is evidence of limited help-seeking and underutilisation of mental health services among South Asians. This includes limited utilisation of British National Health Services talking therapies.^15 16^

Apart from the recognised need to provide equity in healthcare through inclusion of ethnic minorities in research, there are numerous other benefits to increased research participation by this population. For researchers, participation by South Asian communities enables accuracy of subgroup analyses pertaining to ethnicities. It also allows for generalisability of research findings.^17 18^ Evidence suggests South Asian communities have increased predisposition to specific diseases such as diabetes, asthma and cardiovascular disease.^19^,^21^ In the clinic, there is therefore a growing need to tailor treatments as a one-size-fits-all approach does not encompass individual differences in responses to treatment, leading to unsuccessful outcomes. Therefore, understanding research participation motivations will undoubtedly aid the development of effective and innovative treatment options.

The limited inclusion of South Asian populations in mental health research is evidenced by a recently updated review on psychosocial barriers and facilitators to patient recruitment.^22^ It highlighted that motivations for research participation extend beyond the individual to include social and normative influences. The review did not specifically include South Asian communities in the final extraction, although 7% of included studies involved ethnic minorities, which supports the need for more studies in our population of choice. Moreover, only 2.8% of extracted mixed-methods papers dealt with mental illnesses in this review.^22^ Given the unique sociocultural, communication and stigma-related barriers faced by South Asian communities, understanding these motivating factors is essential for developing more inclusive research. However, as the scale of this issue of low South Asian participation in mental health research studies remains undefined, it is difficult and perhaps premature to draw firm conclusions in this area.

The general reasons cited for low participation rates among ethnic minorities include cultural and communication barriers, stigma as well as costs associated with translation of materials. Efforts to tackle these low participation rates have led researchers to adopt more inclusive and participatory methods.^23^ Studies from renal and dementia research suggest that the cultural and communication barriers can be overcome through translation of materials into languages such as Urdu, Bengali and Hindi, as well as involving interpreters to explain study content. Training, specifically cultural sensitivity training at all stages of research planning and dissemination, is advised by experts in the field.^24^ Furthermore, the involvement of expertise from ethnic minority researchers is encouraged to aid participation.^4 6^ Taken together, current literature suggests there exists a gap in learning about the application of these methods for South Asian populations in mental health contexts. This gap persists despite UK healthcare policy imperatives to reduce health inequalities among disadvantaged groups with mental health problems.^25^ Our review responds to these priorities by assessing the means to better understand and address barriers to research participation among people from South Asian communities with mental health problems.

### Review aim

This study aims to systematically review and synthesise qualitative evidence from mental health research studies about participation, motivation and barriers to recruitment of South Asians in high-income countries. We wish to understand community members’ experiences of being approached to take part in research as well as their decision making whether or not to participate. In particular, we wish to gain insight into barriers to and facilitators of participation for these groups, that is, the strategies that encourage or hinder members of the South Asian community to take part in research.

## Methods

This review protocol is reported (PROSPERO registration: CRD42025626382) in line with the Preferred Reporting Items for Systematic Review and Meta-Analysis Protocols (PRISMA-P) 2015 statement,^26^ including relevant elements from the adapted PRISMA checklist for reporting systematic reviews of qualitative (and quantitative) evidence.

### Eligibility criteria

A systematic search of qualitative and mixed methods studies will be performed. Scoping and other types of reviews other than qualitative reviews will be excluded. We will include qualitative studies and mixed-methods studies with a qualitative element. This will include studies that include a qualitative design including phenomenology, meta-ethnography, action research, narrative and descriptive qualitative research. Studies and theses using qualitative methods, such as observations, individual and focus group interviews, will be included.

This review focuses on South Asian minority populations who have been approached or invited to take part in mental health research. Table 1 lists full inclusion and exclusion criteria. The SPIDER (Sample, Phenomenon of Interest, Design, Evaluation type, Research type) framework was used to structure the inclusion and exclusion criteria.

**Table 1:** Inclusion and exclusion criteria for qualitative synthesis following SPIDER criteria

	Inclusion criteria	Exclusion criteria
Sample (S)	Participants of SA backgrounds (Pakistan, India, Bangladesh, Afghanistan, Nepal, Sri Lanka) residing in high-income countries (as defined by the World Bank).^45^ Researchers involved in the identification and recruitment of members of SA communities for participation in MH studies.	Participants of SA background situated in low-income SA countries (ie, where the communities do not constitute a minority) including India, Pakistan, Bangladesh, Afghanistan, Nepal and Sri Lanka. South East Asian (Chinese, Thai, Malay, Japanese, etc). Studies from low- and middle-income countries. Ethnic Arab communities (eg, from Syria, Qatar, Middle East, Saudi Arabia) and Arab-speaking populations (eg, Iraq). Iranian communities. American Indian Alaska Native communities. Native Americans. Aboriginal populations. Hispanic/Latino/Latinx populations. Refugee populations and displaced people.
Phenomenon of Interest (PI)	Studies exploring: MH research participation/representation/under-representation. Barriers to MH research. Facilitators to MH research. Feasibility of taking part in MH research.	Studies on MH service access. Clinical studies. Religion studies. Dementia studies involving members of SA or any other communities.
Design (D)	Qualitative studies including: Interviews. Focus groups. Text-based data collection. Mixed-methods studies with a focus on qualitative outcomes (incl RCTs).	Quantitative studies.
Types of evaluations (E)	SA participant experiences. SA engagement in MH studies. Barriers and facilitators to engaging in MH research for SA communities. Feasibility studies in SAs. Equity and human rights studies.	
Research type (R)	Qualitative studies employing: Thematic analysis. Framework analysis. Ethnography. Grounded theory.	Quantitative studies only, for example, survey responses.

MH, mental health; RCTs, randomised controlled trials; SA, South Asian.

The review will include those individuals who agreed (consented) to take part in a study as well as those who declined participation, but whose opinions were collated. We will also focus on the perspectives of individuals involved in recruiting South Asian communities into studies on mental health, that is, researchers, academics and healthcare professionals. Thus, studies detailing representation, under-representation and feasibility studies involving qualitative methods and outcomes will be included. Randomised controlled trials reporting qualitative data, such as process evaluations, with a distinct focus on mental health in South Asian communities will be included. The outcomes from these will be useful for data extraction in this review. Excluding these may otherwise yield very few papers for final extraction. General research participation studies will be excluded, as these rarely focus on mental health research participation. Similarly, those conducted in South Asians in other research areas (dementia, cancer, diabetes) will be excluded unless there are mental health outcomes reported alongside findings. Papers focusing on South Asians’ access to services and service use will be excluded due to the lack of link to research participation.

### Search strategy

The aim of the searches will be to systematically identify literature on barriers and facilitators to research participation in mental health trials in individuals from South Asian backgrounds. Comprehensive searches of electronic databases will be undertaken. An information specialist (HF) will develop an initial search strategy in Ovid MEDLINE with input from the review team. The strategy will include terms for various concepts with a choice of subject headings and free-text terms. The following five concepts will be included:

1. South Asia (encompassing terms for countries, languages and religions).
2. Ethnic and racial minorities.
3. Mental health conditions.
4. Barriers and facilitators to participation.
5. High-income countries.

The MEDLINE strategy will be adapted as necessary for the other databases and sources searched. No restrictions in terms of study design were applied to any of the searches. Searches were not limited by date but were restricted to English language. The following databases will be searched to identify relevant studies: MEDLINE, EMBASE, PsycINFO, Health Management Information Consortium, Social Policy and Practice, Cochrane Database of Systematic Reviews, Cochrane Central Register of Controlled Trials, Social Sciences Citation Index, Science Citation Index Expanded and ProQuest Dissertations and Theses Citation Index.

All references will be deduplicated in EndNote V.21 (EndNote, 2024; Clarivate)^27^ with the aim of removing further duplicates in Covidence (Veritas Health Innovation, Melbourne, Australia).^28^ Covidence is an online software tool designed to streamline the process of conducting systematic reviews. We will perform forward and backward citation searching with Citationchaser^29^ using all eligible records identified through full text screening.

### Study screening and selection

Titles and abstracts of records will be screened against the inclusion and exclusion criteria by two researchers (NAK, HA) independently and in duplicate. Additional team members will aid the process of title and abstract screening. The title and abstract screening in Covidence employs a machine learning algorithm (active learning) to identify patterns in the research team’s previous screening behaviour. This function displays studies that are most likely to be included first. As more studies are screened (minimum 25 studies, of which 2 must have been included and 2 excluded), the predictive accuracy increases leading to efficiency gains.^30^ Uncertainty about inclusion of titles and abstracts will be resolved through discussion between the two main researchers (NAK, HA). Following full-text screening, the reasons for excluding papers will be recorded and summarised in a PRISMA flow diagram.

The information specialist (HF) will perform forward and backward citation searches of eligible records identified during full-text screening. We will use the Citationchaser (Shiny app)^29^ tool for citation searching. Backward citation refers to the examination of reference lists cited within an article to identify other relevant works stemming from it. In contrast, forward citation search involves searching for papers that have cited the article, to enable the capturing of new ideas or engagement stemming from it.^31^ This supplementary searching process allows us to review additional records that were not captured in the database searches.

### Data extraction and synthesis

The following data will be captured using an Excel sheet:

- Basic study information (authors, title, year(s) of data collection, year of publication).
- Brief summary of the study’s focus, phenomena of interest and theoretical/philosophical basis.
- Study design (including sampling procedures) and description of qualitative methods used (eg, interviews, observation, document analysis).
- Description of the study setting.
- Description of participants (gender, age, socioeconomic status).
- Thematic results.
- Salient discussion points.

The results of included studies, including quotes and tables, will be imported into NVivo software (V.14; Lumivero).^32^ The data from included studies will be synthesised using meta-ethnographic approaches, in line with previous literature^33^ and adaptations of the original approach for utility in syntheses of qualitative literature in healthcare research.^34 35^

Meta-ethnography was selected over other synthesis methods, such as thematic synthesis or meta-aggregation, because it enables an interpretative rather than merely aggregative approach.^36^ This method allows for the ‘translation’ of studies into one another to create new conceptual understandings.^33 37^ This is particularly crucial for this review, as we aim to explore the complex meaning-making processes and cultural nuances that influence South Asian participation, rather than simply listing barriers.

This process involves three levels of ‘construct’, as defined by Britten *et al*^37^:

- First-order constructs: Primary data, participant quotes and observations.
- Second-order constructs: Authors’ (metaphorical) interpretations of primary data.
- Third-order constructs: The reviewers’ new interpretations of original authors’ interpretations, based on their analysis of first-order and second-order constructs.

Full-text papers will be evenly split between two main reviewers (NAK, HA) who will independently extract first-order and second-order constructs. To ensure transparency and rigour, the team will pay close attention to positionality throughout this process. We acknowledge that our interpretation of the data is influenced by our own backgrounds. Specifically, the lead researchers (NAK, HA) bring a bilingual and sociocultural lens to the analysis. We will document how these positionalities shape our interpretation through the use of reflexive memos recorded during the data extraction and synthesis phases. Additionally, a process of double coding will be employed where primary data is mapped against second-order constructs, followed by discussion in team meetings. Thus, several iterations will make up this process until a ‘line of argument’ synthesis is agreed. This synthesis will consider similarities and differences in the studies and represent third order constructs. A table of findings will be made based on agreements and will be used in exploratory subgroup analysis. This process has previously been described in detail by Coventry *et al*.^38^ The review team will also consider employing other methods which might be appropriate to express the synthesised statements, such as conceptual diagrams.

### Assessing methodological limitations

To ensure trustworthiness and richness of the synthesis, each individual review finding will be assessed against the four domains of the Grading of Recommendations Assessment, Development and Evaluation-CERQual (Confidence in the Evidence from Reviews of Qualitative research): relevance, coherence, methodological limitations and adequacy of data. Two reviewers (NAK, HA) will decide whether there are: no or very minor concerns; minor concerns; moderate concerns; or serious concerns for each of the four components.

### Assessing confidence in the cumulative evidence

Based on these assessments, the research team will decide on confidence in the evidence supporting each review statement. Confidence can be judged as very low, low, moderate or high. High confidence means that the phenomenon of interest is unlikely to significantly differ from our analytical themes. All findings start with a high confidence grading and are subsequently downgraded on noting concerns relating to any of the CERQual domains. The final assessment of confidence will be based on consensus and discussion among the review team.

### Ethics and dissemination

Ethical approval is not required as this study is a synthesis of published data.

### Patient and public involvement

Patients or the public were not involved in the design, or conduct, or reporting, or dissemination plans of our research.

### Discussion of existing literature

Identification of barriers to take part and strategies to facilitate participation of South Asian communities in health and social care research has been extensively reported in the last few decades. This evidence is supported by numerous systematic reviews^39^ and reports, such as an extensive report by Hussain-Gambles^40^ which details participants, healthcare providers and lay members’ experiences of clinical trial participation. The report suggests that reasons for South Asian communities to opt in or out of research participation share similarities with the majority population, for example, that relating to mistrust and influence of socioeconomic status. Consequently, efforts to increase participation by minority groups and other underserved groups have been an area of priority in UK research trials. An example of this strategy is the National Institute for Health and Care Research (NIHR)-Innovations in Clinical Trial Design and Delivery for the Under-served (INCLUDE) Ethnicity Framework (2020) which helps trialists design inclusive trials for ethnic minority groups.^41^

A gap in the literature exists regarding the intersection of South Asian identity and the added vulnerability of mental health concerns. We need to understand the factors at play when these identities converge. The context in which this group of people exists, as well as their decision making around research participation, is of interest in this qualitative review. Waheed *et al*^39^ suggested effective recruitment methods for this population. These include selecting ethnically dense geographical sites and using non-health recruitment sources. However, arguably, these recommendations could apply to any underserved or majority community. More specific themes around barriers have been listed in a small-scale RCT, such as family involvement and availability of interpreters, by incorporating views of field researchers.^42^ Incorporating the views of healthcare practitioners, participants and lay people is a welcome addition. However, the current literature remains scarce. Consequently, it may be premature to claim a substantial understanding of the motivations and barriers to mental health research participation.

Thus, it is a strength of this review to allow author reflexivity from researchers with South Asian bilingual backgrounds (NAK, HA), as well as involving them in the entire process leading to publication, such as screening of abstract, titles and full-texts, drawing out constructs and discussing these. Applying cultural knowledge of the belief systems and values of South Asian communities is necessary and will aid interpretation from a sociocultural lens. Supported by field expertise from the remaining authors, the third order constructs in meta-ethnographic analysis will thus aim to be both nuanced and novel. Based on anecdotal information (NIHR Award 156425, University of Hertfordshire),^43^ this issue does not seem uncommon despite the best intentions of mental health researchers to encourage participation from South Asian and other ethnic minority communities.^44^ Therefore, an understanding of these experiences, and what they mean for future research participation on mental health trials, is required.

### Dissemination plan

In summary, this review will provide enlightened insight into what is needed for researchers and practitioners to advance the involvement of South Asian populations into mental health research, by analysing the voices of those often unheard. By systematically synthesising these voices, we aim to move ‘beyond barriers’, shifting the focus from a simple checklist of obstacles towards a deeper conceptual understanding of the structural and intersectional drivers of exclusion. Findings will be disseminated through publication in a peer-reviewed journal and presentation at relevant conferences.
