# Supplementary material for: Beyond barriers: a qualitative evidence synthesis protocol of South Asian involvement in mental health research in high-income countries
Source: BMJ Open. 2026 Feb 12;16(2):e109567. doi: 10.1136/bmjopen-2025-109567 (PMC12911795; doi:10.1136/bmjopen-2025-109567)
Supplement: Supplementary file 1 [file bmjopen-2025-109567.reviewer_comments.pdf]

PEER REVIEW HISTORY

BMJ Open publishes all reviews undertaken for accepted manuscripts. Reviewers are asked to complete a checklist review form and are provided with free text boxes to elaborate on their assessment. These free text comments are reproduced below.

ARTICLE DETAILS

Title (Provisional)

Beyond barriers: a qualitative evidence synthesis protocol of South Asian involvement in mental health research in high-income countries

Authors

Khan, Nilam Akhtar; Coventry, Peter; Ali, Habiba; Fulbright, Helen; Brown, Jennifer V E

VERSION 1 - REVIEW

**Reviewer**	**1**
**Name**	**Ramos-Silva, Alice**
**Affiliation**	**Federal University of Rio de Janeiro, Pharmacy School**
**Date**	**28-Oct-2025**
**COI**	**None**

Overall impression

This is a well-conceived and carefully prepared study protocol, addressing an important and often neglected topic — the underrepresentation of South Asian communities in mental health research. The paper is clear, methodologically sound, and adheres to the PRISMA-P and ENTREQ guidelines. I believe it will make a meaningful contribution to the field of health equity and qualitative evidence synthesis.

That said, I think the manuscript would benefit from some adjustments to improve its conceptual framing and flow. Some sentences are too dense or descriptive, and a few sections could integrate more reflexivity and theoretical grounding, especially regarding intersectionality and epistemic justice.

Overall, I consider this a strong and publishable protocol, pending minor revisions.

Major comments

Conceptual framing – intersectionality and epistemic exclusion

The introduction focuses well on the epidemiology and participation rates, but it could use a stronger conceptual base. For example, framing the issue not only as “barriers to inclusion”

but also as forms of epistemic exclusion would better connect this work with current debates in decolonial and equity research.

→ Suggestion: Include a brief paragraph explaining why underrepresentation is not only methodological but also ethical and epistemic.

Justification for the meta-ethnographic approach

The methods section is solid, but I think you could expand a bit more why meta-ethnography was chosen over other synthesis methods (e.g., thematic synthesis, meta-aggregation). Right now, it reads a bit procedural.

→ It would help to explain that meta-ethnography allows exploring meaning-making processes and cultural nuances that are central to this topic.

Reflexivity section

The reflexivity statement is very good, but it feels detached from the main text. I would recommend integrating reflexivity earlier (perhaps in “Data extraction and synthesis”), showing how the team’s positionality will actually influence analysis (e.g., through reflexive memos, double coding, etc.).

→ This would make the review more transparent and credible.

Discussion – move beyond barriers

The discussion is interesting, but it repeats the idea of “barriers and facilitators” several times. It would be stronger to reframe this as relational and trust-building dynamics between researchers and participants. The phrase “beyond barriers” is in the title but not fully developed conceptually in the text — maybe the authors could connect the title and discussion more explicitly.

Minor comments

Some long sentences could be simplified for clarity. For example, the paragraph starting with “Apart from the recognised need to provide equity...” could be divided in two or three shorter ones.

Check consistency when using “South Asian communities” vs. “South Asians” (sometimes it alternates).

Table 1 is clear, but the layout in the submitted PDF is quite crowded; maybe consider spacing or shading rows for readability.

In-text citations should be checked for uniformity (some have inconsistent punctuation).

The abstract could highlight more clearly the expected analytical contribution, not just procedural steps.

Language and tone

The English is generally very good, but a few minor grammatical adjustments could make it smoother. For example:

“enable provision of effective mental health care” → better as “enable the provision of more effective mental health care”

“thus UK figures relate to these three groups” → consider “so UK figures mainly refer to these three groups”

“it is difficult and perhaps too early to draw conclusions from this particular research area” → could be “it is difficult, and perhaps premature, to draw firm conclusions in this area.” But these are small issues, not major.

VERSION 1 - AUTHOR RESPONSE

Reviewer 1 Comments to Authors:
a) Major comments
Conceptual framing – intersectionality and epistemic exclusion: The introduction focuses well on the epidemiology and participation rates, but it could use a stronger conceptual base. For example, framing the issue not only as “barriers to inclusion” but also as forms of epistemic exclusion would better connect this work with current debates in decolonial and equity research. → Suggestion: Include a brief paragraph explaining why underrepresentation is not only methodological but also ethical and epistemic.
We appreciate this insight. We have added a paragraph to the Introduction to conceptually frame underrepresentation not merely as a methodological issue, but as a form of epistemic exclusion. We have linked this to the ethical imperative of ensuring that knowledge production reflects the diverse lived experiences of these communities.
The paragraph reads as follows:
Current debates in decolonial and equity research highlight that underrepresentation is not merely a methodological oversight but a form of epistemic exclusion (Fricker, 2007; Godrie et al., 2020). By consistently failing to include diverse voices, the research community risks perpetuating a narrow understanding of mental health that privileges dominant cultural norms (Smith, 2021). Addressing this is not only a matter of recruitment targets but an ethical and epistemic imperative to ensure that knowledge production accurately reflects the lived realities of South Asian communities.
Justification for the meta-ethnographic approach: The methods section is solid, but I think you could expand a bit more why meta-ethnography was chosen over other synthesis methods (e.g., thematic synthesis, meta-aggregation). Right now, it reads a bit procedural. → It would help to explain that meta-ethnography allows exploring meaning-making processes and cultural nuances that are central to this topic.
We have expanded the Methods section to explicitly justify the choice of meta-ethnography. We have clarified that this approach was selected over aggregative methods (like thematic synthesis) because it facilitates the translation of studies to explore the complex meaning-making processes and cultural nuances that are central to the intersection of South Asian

ethnicity and mental health.
The added paragraph reads as such:
Meta-ethnography was selected over other synthesis methods, such as thematic synthesis or meta-aggregation, because it enables an interpretative rather than merely aggregative approach (Barnett-Page and Thomas, 2009). This method allows for the 'translation' of studies into one another to create new conceptual understandings (Noblit and Hare, 1988; Britten et al., 2002). This is particularly crucial for this review, as we aim to explore the complex meaning-making processes and cultural nuances that influence South Asian participation, rather than simply listing barriers.

Positionality: The authors should be more explicit about their positionality (lines 43-46 mention "socio-cultural lens" and "meta-synthesis"), showing how the team's positionality will actually influence analysis (e.g., through reflexive memos, double coding, etc.). This would make the review more transparent and credible. We agree that transparency regarding positionality is crucial. We have expanded the section on data extraction and synthesis to explicitly describe how the team's positionality—specifically the bilingual and socio-cultural backgrounds of the researchers—will inform the analysis. We have detailed our use of reflexive memos and the process of double coding to ensure rigour and transparency in our interpretations.
This new paragraph can be found under 'data extraction and synthesis' subsection, and reads as follows:
To ensure transparency and rigour, the team will pay close attention to positionality throughout this process. We acknowledge that our interpretation of the data is influenced by our own backgrounds. Specifically, the lead researchers (NK, HA) bring a bilingual and socio-cultural lens to the analysis. We will document how these positionalities shape our interpretation through the use of reflexive memos recorded during the data extraction and synthesis phases. Additionally, a process of double coding will be employed where primary data is mapped against second-order constructs, followed by discussion in team meetings. Thus, several iterations will make up this process until a 'line of argument' synthesis is agreed
Discussion – move beyond barriers: The discussion is interesting, but it repeats the idea of "barriers and facilitators" several times. The phrase "beyond barriers" is in the title but not fully developed conceptually in the text — maybe the authors could connect the title and discussion more explicitly. We have revised the discussion (now 'Ethics and dissemination' and the concluding sections) to move the narrative beyond a simple reiteration of barriers and facilitators. We have explicitly connected the concept of "Beyond barriers" to our discussion, elaborating on how this review aims to provide novel insights into the structural and intersectional factors affecting participation.
Whilst some sections have been deleted in this section to make this connection clear, as well as sentences restructured, the following concluding sentences have been added:
By systematically synthesising these voices, we aim to move "beyond barriers"—shifting the focus from a simple checklist of obstacles towards a deeper conceptual understanding of the structural and intersectional drivers of exclusion. Findings will be disseminated through

publication in a peer-reviewed journal and presentation at relevant conferences.
b) Minor comments
Some long sentences could be simplified for clarity. For example, the paragraph starting with "Apart from the recognised need to provide equity..." could be divided in two or three shorter ones. We have reviewed the manuscript for sentence length and clarity. Specifically, we have split the paragraph starting "Apart from the recognised need..." into shorter, more concise sentences to improve readability.
Check consistency when using "South Asian communities" vs. "South Asians" (sometimes it alternates). We have reviewed the manuscript to ensure consistent terminology is used when referring to "South Asian communities" and "South Asians".
Table 1 is clear, but the layout in the submitted PDF is quite crowded; maybe consider spacing or shading rows for readability. We have adjusted the formatting of Table 1 to improve readability, including the addition of spacing, as well as shading (every other row) to clearly distinguish between rows.
In-text citations should be checked for uniformity (some have inconsistent punctuation). We have thoroughly checked all in-text citations and corrected any inconsistencies in punctuation and formatting, specifically where commas were used instead of semicolons (between in-text references).
The abstract could highlight more clearly the expected analytical contribution, not just procedural steps. We have revised the abstract to explicitly highlight the study's expected analytical contribution, emphasising the generation of new knowledge regarding the intersectionality of mental health and South Asian ethnicity.
Language and tone: The English is generally very good, but a few minor grammatical adjustments could make it smoother.
"enable provision of effective mental health care" → better as "enable the provision of more effective mental health care" We have amended this phrase to "enable the provision of more effective mental health care" as suggested.
"thus UK figures relate to these three groups" → consider "so UK figures mainly refer to these three groups" We have changed this sentence to read "so UK figures mainly refer to these three groups".
"it is difficult and perhaps too early to draw conclusions from this particular research area" → could be "it is difficult, and perhaps premature, to draw firm conclusions in this area." We have revised this sentence to: "it is difficult, and perhaps premature, to draw firm conclusions in this area."

Please note that as a result of the additional paragraphs added in the manuscript, new references have been inserted and included in the reference list. These can be clearly noted in the marked copy of the manuscript.
